# Association between polycystic ovary syndrome and systemic immune inflammation index and other inflammation markers; a case-control study

**DOI:** 10.4314/ahs.v25i3.11

**Published:** 2025-09

**Authors:** Funda Demirel, Inan Ilker Arikan

**Affiliations:** Kocaeli City Hospital

**Keywords:** Polycystic ovary syndrome, systemic immune inflammation index, neutrophil-lymphcyte ratio, platelet-lymphocyte ratio

## Abstract

**Aim:**

The aim of this study was,to determine the relationship between the systemic immune inflammation index-, which is a new inflammatory marker, and other inflammatory markers in patients with polycystic ovary syndrome.

**Methods:**

This case-control study was conducted by exaimining patients who presented at the Gynecology and Obstetrics Outpatient Clinic of Derince Training and Research Hospital between 2018-2022. Inflammatory markers were compared in hemogram parameters of 180 patients with polycystic ovary syndrome and 178 control group subjects. The two groups were compared in respect of hemoglobin(Hb), white blood cell (Wbc), platelet (Plt), neutrophil (Neu), lymphocyte (Ly), red cell distribution width(RDW), main platelet volume (MPV), platelet distribution width (PDW), neutrophil lymphocyte ratio (NLR), platelet lymphocyte ratio (PLR) and systemic immune inflammation index (SII)values.

**Results:**

The hemoglobin, white blood cell, platelet, neutrophil, lymphocyte,neutrophil lymphocyte ratio,and systemic immune inflammation index values were determined to be statistically significantly higher in PCOS patients than in the control group.

**Conclusion:**

Elevated values of inflammatory markers can be detected in patients with polycystic ovary syndrome,- supporting the presence of inflammation.

## Introduction

Polycystic ovary syndrome (PCOS) is a common endocrine disorder that affects 4-21% of women of reproductive age^1^. Generally, PCOS is characterised by anovulation or oligo-anovulation, excess androgen, and polycystic morphology of the ovary^2^.

The clinical symptoms of PCOS are extremely complex and the exact etiology is still not clear. The components of the diagnostic criteria still remain controver-sial while ethnic differences and variations in clinical features during the life course of women result in a diagnostic challenge^3^. Studies have shown that there could be many factors in PCOS etiology, sggesting that there could be potential associations with reactive oxygen species (ROS)^4^, inflammatory reactions^5^,^6^, genetic predisposition^7^, embryonic over-exposure to androgens^8^, or an unhealthy lifestyle. There are also many findings that low-grade inflammation could be both a symptom and play an important role as a contributing factor^9^,^10^. An increasing number of studies in recent years have focussed on the critical role of inflammation in PCOS, and have reported that significantly high levels of inflammatory cells have been determined in the peripheral blood of PCOS patients^11^,^12^.

The systemic immune-inflammation index (SII) is a new index that is calculated from lymphocyte, neutrophil, and thrombocyte counts. An increasing number of studies have shown that the SII is a useful index reflecting the inflammatory status and systemic immunity of the human body^13^-^15^. The SII has been reported to be associated with various inflammatory and reproductive disorders such as endometrial cancer and ovarian cancer^16^,^17^.

The aim of this study was to compare a patient group with PCOS, which is accepted as having a base of chronic inflammation, and a healthy control group in respect of inflammatory markers in hemogram parameters, and to determine any differences.

## Method

Approval for this case-control study was granted by the Local Ethics Committee (decision no:142, dated:2022). Patients who presented at the Gynaecology and Obstetrics Polyclinic of Kocaeli Derince Training and Research Hospital between 2018 and 2022 were evaluated for inclusion in the study. A patient group was formed of 180 females diagnosed with PCOS and a control group was formed of 178 healthy females. The control group subjects all had a normal appearance of the ovaries on ultrasound and a regular menstrual cycle. The diagnosis of PCOS was made according to the 2003 Rotterdam criteria as the presence of at least two of the following after discounting other symptoms: oligomenorrhea (menstrual cycle lasting >35 days) or amenorrhea (<2 cycles in the last 6 months); clinical or biochemical hyperandrogenism; appearance of polycystic ovary on ultrasound (stromal volume increase and >10 follicles with diameter of 2-8mm, and pearl necklace appearance along the ovary periphery, overgrown ovaries)^2^. Thus, a total of 358 females in the age range of 18-40 years were included in the study. The exclusion criteria were defined as the presence of hyperprolactinemia, thyroid dysfunction, pregnancy or in postpartum period, congenital adrenal hyperplasia, the use of drugs affecting the hypothalamus-ovarian axis, diabetes developing on the basis of chronic inflammation, rheumatismal disease, inflammatory bowel disease, etc.

In the hemogram parameters, the Hb, Wbc, Plt, Neu, Lym, RDW, MPV, and PDW values were analyzed. The neutrophil lymphocyte ratio (NLR) was calculated as the neutrophil count divided by the lymphocyte count. The platelet-lymphocyte ratio (PLR) was calculated as the thrombocyte count divided by the lymphocyte count. The SII was calculated by multiplying the neutrophil count by the thrombocyte count then dividing by the lymphocyte count (Neu x Plt / Lym)^18^.

### Statistical Analysis

Data obtained in the study were analyzed statistically using SPSS vn. 26 software (Statistical Package for the Social Sciences). Descriptive statistics were stated as mean ± standard deviation (SD), median, minimum, and maximum values for continuous data and as number (n) and percentage (%) for categorical data. Conformity of the data to normal distribution was assessed with the Shapiro Wilks test and box-plot graphs. The Student's t-test was applied in the comparisons of two groups of quantitative data showing normal distribution. The Chi-square test was used in the comparisons of categorical data. Receiver Operating Characteristic (ROC) curve analysis and diagnostic screening tests were used to determine the power of the SII to predict the presence of PCOS. Sensitivity is the ability of the test to identify patients within real patients and specificity is the ability to identify healthy individuals within a group of healthy subjects. Positive Predictive Value (PPV) is the measure of the probability that the case is actually a patient when there is a positive result, and Negative Predictive Value (NPV) is the measure of the probability that the case is actually healthy when there is a negative result.

Results were stated in a 95% confidence interval (CI). A value of p<0.05 was accepted as statistically significant.

## Results

Evaluation was made of a total of 358 females with a mean age of 28.20±6.17 years (range, 18-40 years) who presented at Derince Training and Research Hospital between 2018 and 2022 (Table 1).

**Table 1:** Distribution of Descriptive Characteristics

**Age (years)**	*Mean±SD*	28.20±6.17
	*Median (Min-Max)*	27 (18-40)
**BMI (kg/m^2^)**	*Mean±SD*	26.12±4.70
	*Median (Min-Max)*	25.65 (15.6-47.8)
	**Normal**	148 (41.3)
	**Overweight**	151 (42.2)
	**Obese**	59 (16.5)
**Chronic disease**	**Absent**	358 (100)
	**Present**	0 (0)
**Group**	**PCOS patients**	180 (50.3)
	**Control**	178 (49.7)

The mean BMI value of all the cases in the study was 26.12±4.70 kg/m2 (range, 15.6 -47.8 kg/m2), with 148 (41.3%) evaluated as normal weight, 151 (42.2%) as overweight, and 59 (16.5%) as obese. No statistically significant difference was determined between the groups in respect of the BMI classifications (p>0.05) (Table 2).

**Table 2:** Comparisons of the Descriptive Characteristics according to the Groups

		PCOS (n=180)	Control (n=178)	*p*
**Age (years)**	*Mean±SD*			^a^0.001**
		26.31±4.78	30.11±6.80	
	*Median (Min-Max)*			
		26 (18-38)	30 (18-40)	
**BMI**	**Normal**			***^b^0.102***
		82 (45.6)	66 (37.1)	
	**Overweight**			
		64 (35.6)	83 (46.6)	
	**Obese**			
		34 (18.8)	29 (16.3)	

a *Student's t-test*

b *Pearson Chi-Square Test*

** *p<0.01*

No chronic disease was present in any of the patients. The PCOS patient group comprised 180 (50.3%) patients and the control group was formed of 178 (49.7%) subjects (Table 2).

The mean age of the control group was determined to be statistically significantly higher than that of the PCOS patient group (p=0.001, p<0.01) (Table 2).

The Hb,Wbc, Plt, Neu, and Lym values were determined to be statistically significantly higher in PCOS patients than in the control group (p<0.01 for all). No statistically significant difference was determined between the groups in respect of the RDW and PDW values (p>0.05).

The NLR value was determined to be statistically significantly higher in PCOS patients than in the control group (p=0.009, p<0.01). No statistically significant difference was determined between the groups in respect of the PLR value (p>0.05) (Table 3).

**Table 3:** Comparisons of the Clinical Measurements according to the Groups

		PCOS (n=180)	Control (n=178)	Total	*p*
**HB**	*Mean±SD*	12.94±1.25	12.15±1.3	12.55±1.33	***^a^0.001*** **
**WBC**	*Mean±SD*	8.27±2.53	6.95±1.74	7.61±2.27	***^a^0.001*** **
**PLT**	*Mean±SD*	288.02±63.2	261.25±64.36	274.71±65.08	***^a^0.001*** **
**NEU**	*Mean±SD*	5.07±2.01	4.14±1.27	4.61±1.75	***a0.001*** **
**LY**	*Mean±SD*	2.34±0.72	2.15±0.57	2.24±0.66	***^a^0.007*** **
**RDW**	*Mean±SD*	15.11±2.27	15.17±2.47	15.14±2.37	***^a^0.814***
**MPV**	*Mean±SD*	8.83±1.14	9.02±0.94	8.92±1.05	***^a^0.082***
**PDW**	*Mean±SD*	15.06±2.82	15.61±2.63	15.33±2.73	***^a^0.055***
**NLR**	*Mean±SD*	2.47±2.51	1.96±0.66	2.22±1.86	***^a^0.009*** **
**PLR**	*Mean±SD*	135.15±60.27	126.59±37.37	130.89±50.32	***^a^0.108***
**SII**	*Mean±SD*	691.94±519.56	508.76±164.63	600.86±396.48	***^a^0.001*** **

a *Student's t-test*

** *p<0.01*

**Figure 1:**
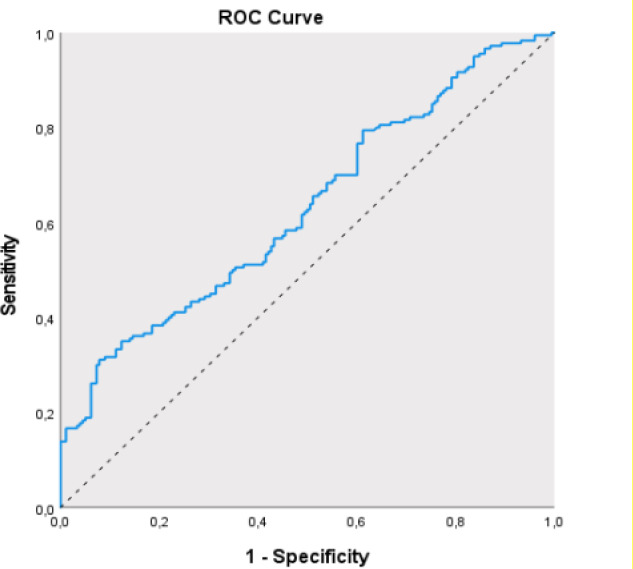
ROC curve of the SII values in the prediction of the presencce of PCOS

The SII value was determined to be statistically significantly higher in PCOS patients than in the control group (p=0.001, p<0.01) (Table 3). For the prediction of the presence of PCOS, a cutoff value of 710.94 for the SII was determined to have 61.11% sensitivity, 91.57% specificity, 78.90% PPV, and 56.80% NPV. The area under the curve (AUC) obtained from ROC curve analysis was 63% and standard error was 2.9%. The cutoff value of 710.94 for SII was determined to be statistically significant in the prediction of the presence of PCOS (p=0.001, p<0.01). The probability of determining PCOS in patients with SII ≥710.94 was determined to be 4.908-fold greater. The ODDS ratio for the SII was 4.908 (95% CI: 0.573-0.687) (Table 4) (Figure 1).

**Table 4:** Results of the Diagnostic Scan and ROC Curve Analysis for SII in the Prediction of PCOS

	Diagnostic Scan					ROC Curve		

	Cut off	Sensitivity	Specificity	Positive Predictive Value	Negative Predictive Value	Area	95% Confidence Interval	*p*
**SII**	***≥710.94***	61.11	91.57	78.90	56.80	**0.630**	0.573-0.687	***0.001***

## Discussion

In this study, the hemogram parameters of PCOS patients and healthy women were compared to determine any differences especially in markers related to inflammation. The study results demonstrated that the inflammation-related markers were higher in the PCOS patient group. The full blood count, which is an easily accessible, low-cost laboratory test often used in clinical practice, provides many data in addition to information about inflammation markers. In this context, Neu, Lym, Wbc, NLR, PLR, and the more recent SII (Neu x Plt/Lym) values are used as inflammattion markers.

In the current study comparisons, the Hb value in the hemogram parameters of the PCOS patient group was determined to be higher than that of the control group. From similar studies in literature, Alhabondi et al. also compared hemogram parameters of a PCOS patient group and a healthy control group and observed no significant difference between the two groups in respect of the Hb value^19^ while Han et al. reported higher Hb values in the PCOS patient group, as in the current study^20^. Hormone levels in women with PCOS affect Hb values. Hyperandrogenism is often seen in patients with PCOS. Testosterone is a hematopoietic hormone and has a dose-dependent stimulating effect on erythropoiesis^21^,^22^. Androgens also affect bone marrow cells mediated by androgen receptors in the bone marrow^23^. The difference in Hb levels is also thought to be due to the decrease in the frequency of menstruation in PCOS patients compared to women without PCOS. This could be the subject of separate future studies.

Also from the hemogram parameters, the Wbc count is one of the parameters in which an increase is observed in inflammation conditions. In studies examining the relationship between PCOS and chronic low-grade inflammation by Papalou et al.^24^, Orio et al.^25^, and Herlihy et al.^26^, the Wbc count was seen to be increased in the PCOS patient group, as in the current study. Similarly in a study of 74 PCOS patients and 51 healthy control group subjects, Ruan et al. determined the WBC count and CRP value, another inflammation marker, to be significantly high in the PCOS group^27^.

Many studies have shown the MPV value from the hemogram parameters to be higher in PCOS groups than in control groups^28^. However, in the current study no significant difference was determined between the groups in respect of the MPV value.

NLR and PLR, which are accepted as inflammatory markers, have been recently investigated in PCOS, which is accepted as an inflammatory process. These markers have been determined to be high in the PCOS patient group in many studies. This was supported in a study by Özay et al.^29^, and Yilmaz et al. reported that the NLR and Neu values were at a high level to make a significant difference in PCOS patients^30^. In the current study, the NLR and Neu value were also found to be significantly high in the PCOS group, but there was no statistically significant difference in the PLR between the groups.

SII is a new inflammatory marker, which is calculated by multiplying the neutrophil count by the thrombocyte count and then dividing by lymphocyte count, and is therefore simple and easily accessible from the hemogram parameters^18^. Although many studies have evaluated inflaammatory markers in PCOS patient groups, evaluation of the relationship between SII and PCOS is a new subject of research. The aim of the current study was to investigate this relationship.

The SII, which is an easily obtained, low-cost, non-invasive marker, was first described by Hu et al. in hepatocellular cancer patients^31^. In subsequent years, it became the subject of research in cancer types such as colorectal cancer^32^, cervical cancer^33^, and lung cancer, and in many diseases that develop on the basis of inflammation such as coronary artery disease and acute ischaemic attack^34^. When calculating the SII, the data obtained from thrombocyte, neutrophil, and lymphocyte counts are combined and thus three different biological pathways are reflected: thrombus formation, inflammatory response, and adaptive immune reesponse^35^,^36^.

In contrast to traditional inflammatory factors, the SII reflects the inflammatory status better, and a series of studies have shown that the SII is more prognostic and preferred for reflecting the inflammatory status^37^,^38^. Studies by Li X et al., which examined the relationship between the SII and in-vitro fertilisation (IVF) results in infertile women with PCOS, supported many studies showing that inflammatory cell concentration was significantly high in the blood of the PCOS patients^39^,^40^, and it was reported that abnormal inflammation led to deterioration in oocyte quality and contributed to infertility^41^.

The results of the current study showed that there was a basis of abnormal inflammation in PCOS patients, and the SII value, showing this inflammation and which is a more prognostic marker, was increased. These results supported that there is a base of chronic inflammation in PCOS patients.

## Conclusion

Inflammation markers such as SII, NLR, and PLR, which can be determined from the full blood count, may be different in patients with PCOS, the basis of which is considered to be chronic inflammation.

The author thanks Arzu Yavuz for her contributions to this study.
